# Value of ultrasound assessment of sarcopenia in patients with diffuse large B-cell lymphoma: a prospective study

**DOI:** 10.2478/raon-2025-0036

**Published:** 2025-07-08

**Authors:** Teng Liu, Junjun Wan, Yangyang Liu, Jing Zhuo, Weijiang Fu

**Affiliations:** Department of Ultrasound, Qilu Hospital of Shandong University, Jinan, China; Department of Respiratory Medicine, the Second Hospital of Shandong University, Jinan, China; Department of Ultrasound, the People’s Hospital of Yinan, Linyi, China; Department of Geriatric Medicine, Qilu Hospital of Shandong University, Jinan, China; Shandong Key Laboratory Cardiovascular Proteomics, Jinan, China

**Keywords:** diffuse large B-cell lymphoma, sarcopenia, ultrasound, muscle

## Abstract

**Background:**

This study investigated the clinical utility of ultrasound in diagnosing sarcopenia in patients with diffuse large B-cell lymphoma (DLBCL), focusing on muscle mass, strength, and physical fitness.

**Patients and methods:**

A prospective analysis was conducted on 167 patients with DLBCL (88 with sarcopenia and 79 without). Muscle thickness (MT), cross-sectional area (CSA), and subcutaneous fat thickness (SFT) were measured using ultrasound at various anatomical sites. Diagnostic efficacy of muscle indices for sarcopenia was assessed using receiver operating characteristic (ROC) curves.

**Results:**

Patients with sarcopenia exhibited significant reductions in MT and CSA across multiple muscle groups, including biceps brachii (BB), vastus intermedius (VI), and rectus femoris (RF) (all p ≤ 0.001). ROC analysis identified RF-CSA as the most effective indicator of sarcopenia, with an area under the curve (AUC) of 0.87, a sensitivity of 86%, and a specificity of 83% at a critical value of 7.08 cm^2^. Multivariate analysis revealed that reduced MT and CSA significantly increased the risk of sarcopenia after adjusting for age, gender, and physical performance.

**Conclusions:**

Ultrasound was a cost-effective and accessible diagnostic tool for identifying sarcopenia in DLBCL patients. Early detection through ultrasound can guide timely interventions and improve clinical outcomes.

## Introduction

Sarcopenia is a clinical syndrome characterized by progressive and systemic decline in skeletal muscle mass and strength. This condition is closely associated with an increased incidence of adverse events and poorer prognosis, consequently leading to a significant rise in patient mortality rates. The diagnosis of sarcopenia is confirmed by the assessment of reduced muscle mass and quality, while poor physical performance signifies severe sarcopenia.^1^ This condition not only increases the risk of falls, fractures, and frailty but also impairs mobility, leading to higher care costs and reduced quality of life.^2^ As research into skeletal muscle metabolism deepens, people’s understanding of sarcopenia extends from age-related factors to tumors, inflammation, hormonal levels, nutritional status, chronic consumptive diseases, and even genetic levels. Sarcopenia offers an objectively measurable phenotype that is easily reproducible, potentially modifiable, and correlated with frailty.^3^ Cancer can cause cachexia and sarcopenia. Previous research on cancer patients has demonstrated a significant incidence of sarcopenia, ranging from 15% to 59%.^4^ Recent studies have indicated that sarcopenia serves as a critical independent risk factor for postoperative complications, chemotherapy toxicity, adverse effects, and poor prognosis in cancer patients, underscoring its significant prognostic relevance.^5–11^ Furthermore, in hematological malignancies, sarcopenia appears to have prognostic implications^12,13^, although evidence of this has not been widely disseminated in published research.

Lymphoma is a group of biologically and clinically heterogeneous neoplastic entities that rank tenth in the most common types of cancer worldwide and eleventh in the causes of cancer-related death with a significant upward trend in the more advanced age groups.^14,15^ Diffuse large B-cell lymphoma (DLBCL) is the most common type of nonHodgkin’s lymphoma, with the incidence rate increasing with age, and the median age at diagnosis is 70 years old.^16,17^ Some studies have found that sarcopenia is associated with a decrease in the survival rate of DLBCL.^18^ Early screening and diagnosis of sarcopenia, followed by appropriate intervention, are essential for patients with DLBCL to manage their condition effectively.

Currently, a range of clinical techniques are employed to assess skeletal muscle mass, including bio-electrical impedance analysis (BIA), dualenergy X-ray absorptiometry (DXA), computed tomography (CT), and magnetic resonance imaging (MRI). CT and MRI are acknowledged as the gold standards for quantifying skeletal muscle content and are extensively utilized in this capacity for this purpose.^19^ Although BIA, DXA, MRI and CT are recommended by the European Working Group on Hypomuscular Disease in the Elderly (EWGSOP) for assessing muscle mass, their widespread adoption is hindered by their substantial costs and operational limitations. Furthermore, the reliability of BIA results is significantly influenced by the patient’s overall hydration status. The clinical utility of CT and MRI is constrained by several factors, including their high costs, the requirement for specialized facilities, and the radioactivity associated with CT scans. Consequently, there is a growing interest in exploring alternative imaging technologies. One such alternative is the utilization of ultrasound.

Recently, ultrasound has gained increasing attention for measuring muscle mass due to its high portability, low cost, no ionizing radiation, high reproducibility.^20^ Particularly in scenarios where CT, DXA, or MRI are challenging to execute, ultrasound emerges as a beneficial modality for evaluating muscle volume and integrity. Muscle thickness (MT) and muscle cross-sectional area (CSA), two common ultrasound parameters for muscle mass, have been widely used in sarcopenia research.^20–23^ However, the cut-off points of ultrasound parameters for diagnosing sarcopenia have not been established. The use of ultrasound to assess sarcopenia requires the establishment of standardized measurement methods, the identification of the optimal muscle groups for evaluation, and the determination of critical values under different populations, clinical environments, and conditions.

Currently, the use of ultrasonography for assessing sarcopenia is increasing, but it has not yet been applied to patients with DLBCL. This study is the first prospective evaluation of the clinical value of ultrasound examination in diagnosing sarcopenia in DLBCL patients. The aim of this study is to assess the thickness and cross-sectional area of upper and lower limb muscles in DLBCL patients using ultrasound, determine the optimal muscle and cut-off values, and explore the practicality of ultrasound measurement in diagnosing sarcopenia in DLBCL patients.

## Patients and methods

### Study population

**FIGURE 1.**
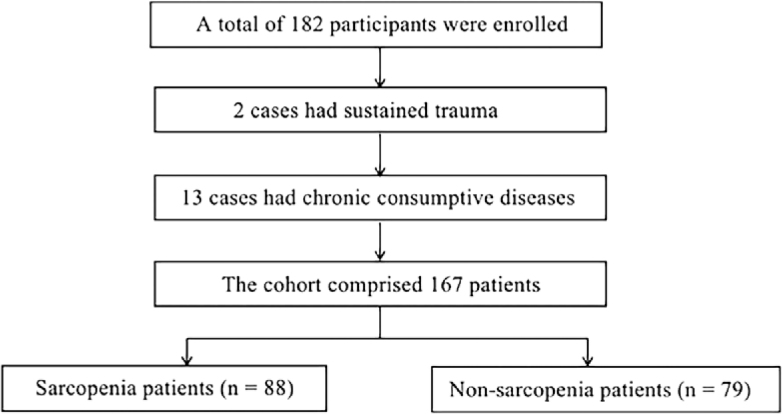
Participant flow chart for study. A total of 167 cases were eventually recruited into the sarcopenia (n = 88) and non-sarcopenia (n = 79) groups for analysis.

The study population of this research is patients with DLBCL who received inpatient care at Qilu Hospital of Shandong University. The research data is sourced from our clinical database, covering the time period from October 2022 to October 2023. Inclusion criteria: (a) Clinically diagnosed with DLBCL; (b) Have complete abdominal CT imaging materials; (c) Able to cooperate with ultrasound examination and skeletal muscle strength tests (including gait speed test, grip strength test). Exclusion criteria: (a) Body impairment, limb trauma, or surgical history; (b) Those with combined neuro-muscular system diseases; (c) Those with combined chronic wasting diseases (such as diabetes, chronic obstructive pulmonary disease, chronic liver disease, hyperthyroidism, etc.). Finally, the cohort comprised 167 patients. They were grouped into sarcopenia (n = 88) and non-sarcopenia (n = 79) groups (Figure 1). The study was approved by the Bioethics Committee of Qilu Hospital of Shandong University (registration number: KYLL-202207-017-1, date 08/10/2022) and was conducted according to the Declaration of Helsinki. All participants were informed in detail about this research and gave their written consent.

### Data collection

We collected demographic and clinical data, including the following variables: age, gender, height, weight, calf circumference (CC), body mass index (BMI), BMI calculation formula is: BMI = weight (kg)/height^2^ (m^2^).

### Diagnosis of sarcopenia

Measurements of total muscle areas (TMAs) were normalized by height (m^2^) for the skeletal muscle index (SMI). The diagnostic criteria for sarcopenia are based on the skeletal muscle index at the third lumbar vertebrae (L3). For diagnosing sarcopenia, existing cut-off values for SMI at level L3 adapted to body mass index (BMI) recommended by Martin et al. were used: SMI < 43 cm^2^/m^2^ for male patients with BMI < 25 kg/m^2^, SMI < 53 cm^2^/m^2^ for male patients with BMI ≥ 25 kg/m^2^ and < 41 cm^2^/m^2^ for females.^4^

### Assessment of muscle strength

Handgrip strength of the dominant hand was measured 3 times using a dynamometer, with an interval of 1 min between each measurement. The mean value was recorded. Low muscle strength was defined as grip strength < 28 kg and < 18 kg for men and women, respectively.^4^

### Assessment of physical performance

All subjects were assessed by a trained physician for 6-m walking speed test. Participants were asked to walk 6 m at normal speed from a moving starting point and the time taken was recorded and the average time of the two trials was analyzed as the result. Low gait speed is defined as < 1.0 m/s. All assessments were conducted in a quiet room dedicated to clinical assessment.^24^

### Muscle ultrasound measurements

Ultrasound data were acquired by a proficient sonographer utilizing a GE Logiq E9 and Philips EPIQ7 high-frequency linear probes. The thickness of the limb muscles, cross-sectional area, and subcutaneous fat thickness were systematically measured. To ensure the consistency and accuracy of the data, the assessment of limb muscles for all patients was conducted by the same professional examiner, who guided the patients to maintain a completely relaxed state during the ultrasound scanning and image recording process. During the scanning process, the patient’s limbs are alternately extended and relaxed to ensure that the muscles reached a state of relaxation. The examiner firmly and vertically positions the ultrasound probe against the skin surface to ensure close contact between the probe and the skin. Simultaneously, an adequate amount of ultrasound gel is used to secure the probe at the measurement site, thereby ensuring the accuracy of the measurement results. Following a 10-minute rest interval, three measurements were taken at the same location, and the final result was determined by averaging the three readings.

**FIGURE 2.**
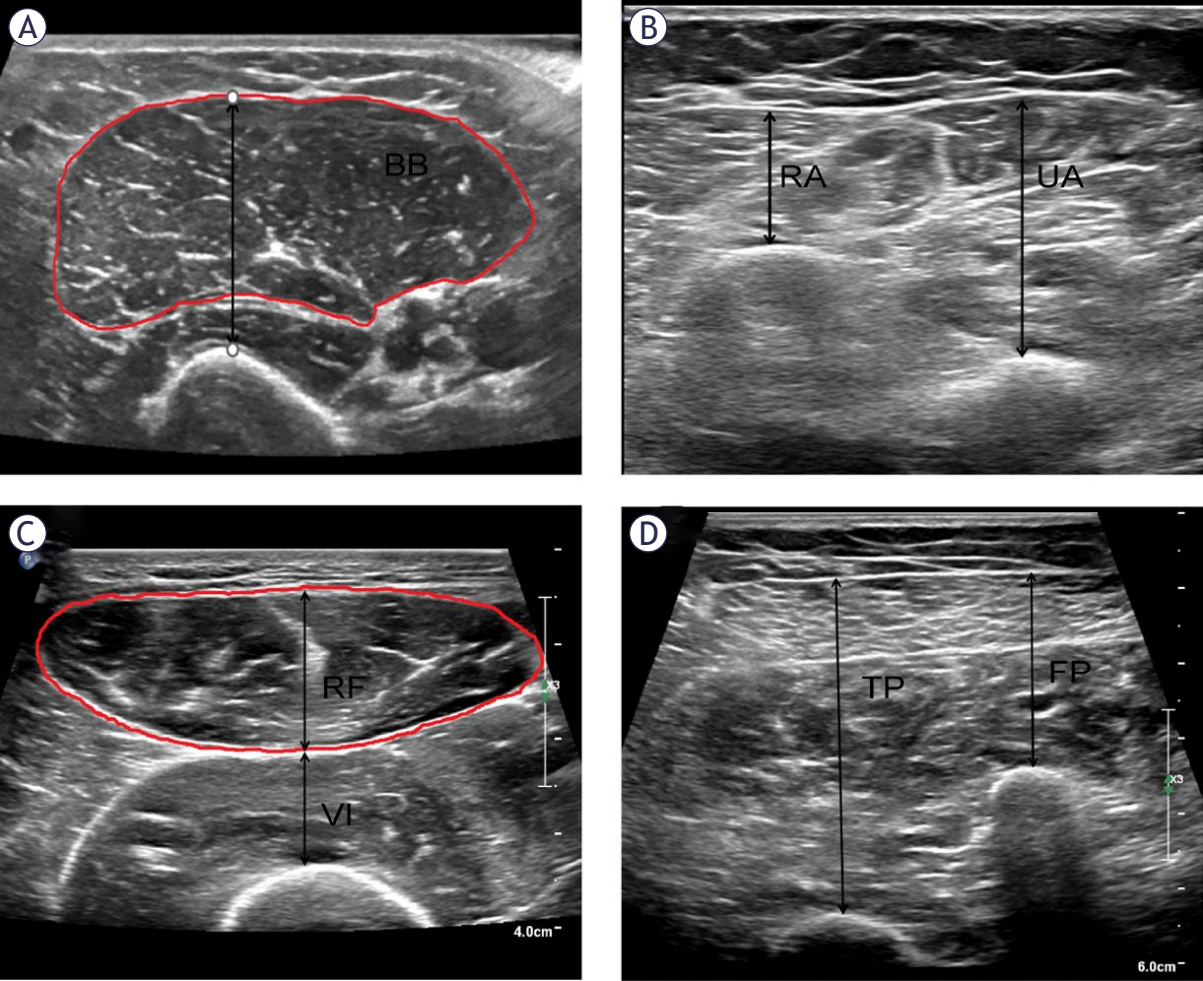
Muscle thickness and cross-sectional area of the extremity muscles. **(A)** Muscle thickness of biceps brachii (BB) and brachialis, cross-sectional area of BB; **(B)** Muscle thickness of radial anterior (RA) muscle and ulnar anterior (UA) muscle; **(C)** Muscle thickness and cross-sectional area of rectus femoris (RF), muscle thickness of vastus intermedius (VI); **(D)** Muscle thickness of tibialis posterior (TP) and fibula posterior (FP).

Upper limb muscles were measured bilaterally with the participants lying supine and arms naturally positioned on either side of the body with palms up. The assessment of the biceps brachii (BB) was conducted at the midpoint between the acromion and the cubital fossa. This position ensures that the probe is perpendicular to the long axis of the upper arm. During the measurement, the following parameters are assessed: MT of BB and brachialis, CSA and subcutaneous fat thickness (SFT) of the BB. For the muscles surrounding the ulna and the anterior radius, the probe is positioned on the forearm, starting from the radial styloid and extending to the proximal one-third of the radial head. At this location, the MT and SFT are measured. Lower limb muscles were measured bilaterally with the participants lying supine and prone separately. To measure the quadriceps femoris (QF), position the probe’s long axis at the midpoint between the superior edge of the patella and the anterior superior iliac spine. Measure were the thickness of the QF (including the rectus femoris [RF] and vastus intermedius), the thigh subcutaneous fat thickness (SFT), and the CSA of RF separately. For the muscle groups at the posterior end of the fibula and tibia, measurements are taken in the proximal third between the medial condyle of the tibia and the medial malleolus (Figure 2).

### Statistical analysis

All analyses were performed using R Statistical Software (Version 4.2.2, http://www.R-project.org, The R Foundation) and Free Statistics analysis platform (Version 1.9, Beijing, China). Quantitative data are presented as means ± standard deviations (SD). For the comparison of means between two groups, the Student’s t-test was used. Chisquare tests were used for categorical variables. Ultrasound measurements of MT and CSA were tested for their ability to predict sarcopenia using receiver operating characteristic (ROC) curves. The overall performance of the ROC analysis was quantified by computing the area under the curve (AUC). ROC analysis was used to determine the optimal sensitivity and specificity of various cutoff values for the prediction of sarcopenia. After adjusting for relevant factors such as gender and age, a series of multiple logistic regression analysis was conducted to identify the association of ultrasound measurement of MT and CSA with sarcopenia. Calculate the adjusted odds ratio (odds ratio, OR) and its 95% confidence interval based on the results of logistic regression analysis. P-values less than 0.05 were considered statistically significant.

## Results

A total of 182 participants were enrolled in the study as potential eligible subjects. According to the exclusion criteria, 2 cases had sustained trauma, 13 cases had chronic wasting diseases, and 15 cases were excluded. Ultimately, this study included a total of 167 individuals (98 males and 69 females), with an average age of 60 ± 14.15 years. All diagnostic procedures for sarcopenia (clinical assessment, grip strength and CT examination) and ultrasound examination were completed within a week, with no adverse events occurring. The demographic characteristics and muscle parameters measured by ultrasound are shown in Table 1.

**TABLE 1.** Patient clinical characteristics at diagnosis and comparison between patients with or without sarcopenia

	Total (n = 167)	Sarcopenia (n = 88)	No sarcopenia (n = 79)	P value
Age (years)	60 ± 14.15	63 ± 14.70	56 ± 12.65	0.002
Gender, n (%)				< 0.001
Males	98 (59)	40 (46)	58 (73)	
Females	69 (41)	48 (54)	21 (27)	
Height (cm)	166.63 ± 8.55	164.69 ± 8.42	168.78 ± 8.23	0.002
Weight (kg)	66.24 ± 11.76	61.33 ± 10.61	71.71 ± 10.54	< 0.001
BMI (kg/m^2^)	23.79 ± 3.17	22.60 ± 3.08	25.12 ± 2.74	< 0.001
CC (cm)	33.50 ± 3.25	32.00 ± 2.69	35.18 ± 3.00	< 0.001
Handgrip strength (kg)	23.19 ± 10.00	18.50 ± 7.51	28.41 ± 9.88	< 0.001
Gait speed (m/s)	1.02 ± 0.29	0.92 ± 0.24	1.14 ± 0.29	< 0.001
SMA (cm^2^)	116.71 ± 27.89	98.86 ± 18.26	136.59 ± 22.89	< 0.001
SMI (cm^2^/m^2^)	41.63 ± 7.57	36.21 ± 4.73	47.67 ± 5.17	< 0.001
Upper arm SFT (cm)	0.30 ± 0.16	0.29 ± 0.14	0.32 ± 0.18	0.23
BB and brachialis MT (cm)	2.29 ± 0.46	2.02 ± 0.38	2.59 ± 0.34	< 0.001
BB CSA (cm^2^)	8.24 ± 2.68	6.67 ± 2.11	9.99 ± 2.10	< 0.001
Forearm SFT (cm)	0.36 ± 0.15	0.35 ± 0.17	0.37 ± 0.14	0.498
UA MT (cm)	3.55 ± 0.49	3.34 ± 0.46	3.79 ± 0.41	< 0.001
RA MT (cm)	1.63 ± 0.36	1.48 ± 0.32	1.79 ± 0.33	< 0.001
Thigh SFT (cm)	0.78 ± 0.36	0.78 ± 0.37	0.78 ± 0.35	0.989
RF MT (cm)	1.54 ± 0.33	1.36 ± 0.30	1.74 ± 0.23	< 0.001
VI MT (cm)	1.45 ± 0.41	1.22 ± 0.34	1.71 ± 0.33	< 0.001
QF MT (cm)	2.99 ± 0.70	2.58 ± 0.59	3.45 ± 0.51	< 0.001
RF CSA (cm^2^)	7.12 ± 1.93	6.01 ± 1.56	8.36 ± 1.51	< 0.001
Lower leg SFT (cm)	0.45 ± 0.19	0.45 ± 0.17	0.45 ± 0.21	0.844
TP MT (cm)	4.87 ± 0.74	4.49 ± 0.62	5.28 ± 0.63	< 0.001
FP MT (cm)	2.39 ± 0.71	2.10 ± 0.61	2.72 ± 0.67	< 0.001

1 BB = biceps brachii; BMI **=** body mass index; CC = calf circumference; CSA = cross-sectional area; FP = fibula posterior; MT = muscle thickness; RA = radial anterior; RF = rectus femoris; QF = quadriceps femoris; SFT = subcutaneous fat thickness; SMA = skeletal muscle area; SMI = skeletal mass index; TP = tibialis posterior; UA = ulnar anterior; VI = vastus intermedius

In this study, there were 88 patients (53%) with sarcopenia, among the participants, with a higher proportion of female patients (54%). As expected, the sarcopenia group had a lower BMI (22.60 vs. 25.12 kg/m^2^, p < 0.001), smaller calf circumference (32.00 vs. 35.18 cm, p < 0.001), handgrip strength (18.50 vs. 28.41 kg, p < 0.001), slower gait speed (0.92 vs. 1.14 m/s, p < 0.001), smaller skeletal muscle area (SMA) (98.86 vs. 136.59 cm^2^, p < 0.001) and SMI (36.21 vs. 47.67 cm^2^/m^2^, p < 0.001) compared to the non-sarcopenia group. Ultrasound measurements revealed that MT of biceps brachii (BB) and brachialis, CSA of biceps brachii (BB), MT of ulnaris anterior (UA) and radialis anterior (RA), MT of vastus intermedius (VI) and QF, MT and CAS of RF, and MT of tibialis posterior (TP) muscle and fibula posterior (FP) muscle were significantly reduced in patients with sarcopenia (p < 0.001).

**FIGURE 3.**
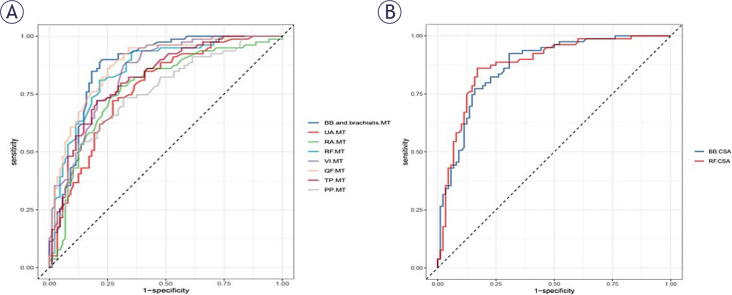
ROC analysis for the prediction of sarcopenia from extremity muscle thickness and cross-sectional area. **(A)** The predictive value of extremity muscle thickness. **(B)** The predictive value of extremity muscle cross-sectional area. BB = biceps brachii; CSA = cross-sectional area; MT = muscle thickness; PP = psoas posterior; RA = radial anterior; RF = rectus femoris; QF = quadriceps femoris; TP = tibialis posterior; UA = ulnar anterior; VI = vastus intermedius

Receiver Operating Characteristic (ROC) curve analysis shows that ultrasonic measurement of limb MT and CSA can predict sarcopenia (Figure 3). Table 2 presents the results of the ROC analysis using ultrasonic measurement to predict sarcopenia, showing a significant correlation. Among them, the AUC for the CSA of the RF was the highest, at 0.8759. The optimal cut-off value for the RF-CSA to predict sarcopenia was 7.08 cm^2^, with a sensitivity of 86% and a specificity of 83%. The AUC values for the MT of BB and brachialis, UA, RA, RF, VI, QF, TP and CSA of the BB ranged from 0.7713 to 0.8746. ROC curve analysis shows that the MT (Figure 3A) and CSA (Figure 3B) of the limb muscles measured by ultrasound can predict sarcopenia.

**TABLE 2.** ROC analysis results for the prediction of sarcopenia from ultrasonographically measured muscle thickness and cross-sectional area

	AUC(95%CI)	Cut-off	Specificity	Sensitivity	Accuracy
BB and brachialis MT (cm)	86.87% (81.28% ~ 92.47%)	2.22	0.77	0.90	0.83
BB CSA(cm^2^)	86.82% (81.41% ~ 92.23%)	7.46	0.69	0.92	0.80
UA MT(cm)	77.13% (70.04% ~ 84.22%)	3.55	0.73	0.72	0.72
RA MT(cm)	77.44% (70.14% ~ 84.73%)	1.56	0.69	0.78	0.74
RF MT(cm)	85.34% (79.56% ~ 91.12%)	1.58	0.78	0.81	0.80
VI MT(cm)	84.07% (78.24% ~ 89.9%)	1.38	0.67	0.89	0.77
QF MT(cm)	87.46% (82.17% ~ 92.75%)	2.92	0.74	0.87	0.80
RF CSA(cm^2^)	87.59% (82.16% ~ 93.02%)	7.08	0.83	0.86	0.84
TP MT(cm)	81.77% (75.42% ~ 88.12%)	4.90	0.80	0.72	0.76
FP MT(cm)	75.75% (68.44% ~ 83.06%)	2.20	0.68	0.73	0.71

1 AUC = area under the curve; ROC = receiver operating characteristic

1 BB = biceps brachii; CSA = cross-sectional area; FP = fibial posteriot; MT = muscle thickness; RA = radial anterior; RF = rectus femoris; QF = quadriceps femoris; TP = tibialis posterior; UA = ulnar anterior; VI = vastus intermedius

Both univariate and multivariate analyses were performed for sarcopenia predictive ultrasound muscle thickness measurements, using the cut-off values from ROC analyses (Table 3). After adjusting for age, gender, BMI, gait speed, grip strength, and calf circumference, the risk of sarcopenia in cases with skeletal muscle parameters less than the cut-off value was 9.47-126.27 times higher than in cases with values greater than the cut-off value.

**TABLE 3.** Odds ratios and 95% confidence intervals for the prediction of sarcopenia from ultrasound parameters

	Non-adjusted		Model I		Model II	
	OR (95% CI)	P value	OR (95% CI)	P value	OR (95% CI)	P value
BB and brachialis MT	28.32 (11.74~68.27)	< 0.001	107.93 (12.01–970.38)	< 0.001	126.27 (12.06–1321.54)	< 0.001
BB CSA	26.07 (10.13–67.11)	< 0.001	20.56 (5.85–72.29)	< 0.001	16.55 (4.5–60.85)	< 0.001
UA MT	6.91 (3.5–13.63)	< 0.001	3.42 (1.42–8.22)	0.006	2.7 (1.04–6.99)	0.041
RA MT	7.42 (3.7–14.89)	< 0.001	3.74 (1.65–8.46)	0.002	3.17 (1.35–7.45)	0.008
RF MT	15.49 (7.26–33.05)	< 0.001	8.19 (3.55–18.93)	< 0.001	6.28 (2.64–14.98)	< 0.001
VI MT	13.61 (6–30.87)	< 0.001	10.68 (4.13–27.58)	< 0.001	9.52 (3.47–26.12)	< 0.001
QF MT	19.5 (8.62–44.1)	< 0.001	13.04 (5.25–32.38)	< 0.001	11.08 (4.23–29.04)	< 0.001
RF CSA	30.08 (12.92–70.05)	< 0.001	15.06 (6.03–37.6)	< 0.001	13.26 (5.11–34.43)	< 0.001
TP MT	9.41 (4.64–19.08)	< 0.001	4.58 (2–10.45)	< 0.001	3.65 (1.53–8.73)	0.004
PP MT	5.62 (2.88–10.96)	< 0.001	3.72 (1.66–8.34)	0.001	2.85 (1.23–6.6)	0.014

1 BB = biceps brachii; CI = confidence interval; CSA = cross-sectional area; HR = hazard ratio; MT = muscle thickness; OR = odds ratio; PP = psoas posterior; RA = radial anterior; RF = rectus femoris; QF = quadriceps femoris; TP = tibialis posterior; UA = ulnar anterior; VI = vastus intermedius

1 Model I was adjusted for demographic features, including age, gender and BMI;

1 Model II was adjusted for demographic features and physical performance, including age, gender, BMI, gait speed, handgrip strength and calf circumference.

## Discussion

Currently, human body composition assessment is an emerging research filed in general medicine, especially in radiology and oncology. Normal populations begin to gradually lose muscle mass from the age of 50.^25^ Sarcopenia is a complex phenomenon with multifactorial aetiologies. In cancer, it can be partially explained by the intricate hormonal network, including anabolic and catabolic factors such as protein synthesis, proteolysis, neuromuscular integrity, and muscle fat content, which are induced by the tumour cells or by the host response. Ultimately, these factors lead to the depletion of skeletal muscle mass and can affect the distribution of chemotherapeutic drugs.^1,26^ Measurements of body composition for oncology populations have received increasing attention in recent years, and the association of tumour malignancy and baseline muscle mass loss with poor clinical outcomes has been well described in patients with a variety of solid tumours and malignant haematological disorders.^27,28^ Notably, most studies have tested the prognostic significance of sarcopenia but not its predictive role.

The idea of diagnosing sarcopenia through radiological examinations is innovative and a step in the right direction. In the early stages, researchers utilized DXA to assess skeletal muscle mass; however, the precision of this method had certain limitations. In light of this, CT and MRI technologies were swiftly adopted and extensively applied for such measurements. Although CT and MRI are considered the gold standard for assessing muscle mass, their use is limited in some patients due to high costs, time-consuming examinations, limited space of the scanners, and limited availability and accessibility. Ultrasound shows good effectiveness in estimating muscle mass compared to MRI, CT, and DXA.^1,29,30^ Since the target patients are often fragile, elderly, and immobile, the imaging technique must be easily accessible both geographical and physical terms, in which ultrasound technology clearly surpasses the previous methods. Ultrasound is potentially valuable in quantifying muscle in patients with sarcopenia, allowing sensitive measurement of muscle fat degeneration which is an important indicator of muscle mass. The non-invasiveness and convenience of ultrasound endow it with significant advantages and a broad prospect in clinical applications. Although research on the use of ultrasound in patients with DLBCL is still relatively limited at present, existing studies have demonstrated that ultrasound can effectively assess changes in muscle mass among cancer patients. Therefore, ultrasound technology is also expected to play an important role in patients with DLBCL, providing strong support for clinical diagnosis and treatment.

Skeletal muscle index and grip strength are universally recognized methods for measuring muscle mass and strength.^31–33^ There are fewer previous studies using ultrasound to assess sarcopenia in patients with lymphoma. This study explores the application value of ultrasound measurement in patients with diffuse large B-cell lymphoma by assessing MT, CSA, and SFT of the extremity muscles using ultrasound. This study’s results show that we have identified the prevalence of sarcopenia in a cohort of patients with diffuse large B-cell lymphoma to be 53%, which is consistent with previous research findings (23.9 to 55.6%).^34^ Compared to non-sarcopenic subjects, the sarcopenic group showed decreases in body mass index, calf circumference, grip strength, and gait speed. Ultrasonographic measurements of MT of BB and brachialis, CSA of BB, MT of the ulnar and radius anterior groups, MT of the vastus medialis and rectus femoris, CSA of the rectus femoris, and MT of TP and FP were significantly reduced in sarcopenic patients (P < 0.001). Ultrasonic measurement of MT and CSA is a visual method for assessing muscle mass loss, and it is believed that muscle mass loss leads to a decline in muscle strength. According to the research results, for patients with diffuse large B-cell lymphoma, especially for those who cannot receive BIA clinically, ultrasonic assessment of muscle mass is an effective and feasible technique.

The statistical analysis conducted in this study showed that compared to non-sarcopenic individuals, those with sarcopenia had lower MT and CSA. Determining the cut-off value for ultrasound measurement of sarcopenia is important in order to provide a quick and easy clinical judgement of muscle mass and to help identify the at-risk population who require timely intervention. Through our study, we were able to identify that the CSA of RF has the highest AUC for sarcopenia, with the optimal critical value for CSA of RF being 7.08 cm^2^. Possible reasons for this are that sarcopenia initially affects the muscles of the abdomen and the front of the thighs, and RF-CSA can sensitively reflect the loss of muscle mass. Studies have reported that using a similar ultrasound protocol to assess the CSA of healthy elderly individuals yielded a value of 4.63 cm^2^, the RF-CSA of elderly COPD patients was 3.48 cm^2^,^35^ the CSA of hemodialysis patients was 1.70 cm^2^,^32^ and the RF-CSA in male and female patients with sarcoidosis was 8.69 mm^2^ and 6.54 mm^2^, respectively. This indicates that there are significant differences in the cut-off values for the CSA of RF among different populations and diseases, hence further differentiation is needed among these different groups. Statistical analyses performed in this study showed that the risk of sarcopenia is increased several-fold in individuals with lower muscle thickness and cross-sectional area, as shown by the adjusted odds ratio.

Chronic inflammation, as a prevalent comorbidity in cancer patients, can compromise the accurate assessment of sarcopenia through multiple pathophysiological pathways. The resulting fluid retention and chronic edema may introduce significant bias into ultrasound measurements. Current evidence indicates that in patients with chronic inflammatory diseases (such as rheumatoid arthritis and chronic heart failure), edema can lead to a systematic 15%-20% overestimation of muscle ultrasound parameters. This phenomenon is particularly pronounced in lymphoma patients, where characteristic lymphatic drainage dysfunction and inflammatory responses create a vicious cycle that exacerbates fluid retention. Notably, the current EWGSOP guidelines have not yet incorporated a correction system for muscle assessment in the context of inflammation-related edema - an oversight that underscores the clinical urgency for establishing standardized measurement protocols. Future multicenter studies are urgently needed to systematically evaluate the stability of ultrasound indicators under varying fluid-load conditions, thereby enhancing the generalizability of this technology in diagnosing cancer-related sarcopenia.

This study has certain limitations. Firstly, this study is a single-center investigation with a relatively small sample size, which thereby limits the generalizability of our findings to a broader national population. Secondly, this study analyzed only the most common type of lymphoma, i.e., diffuse large B-cell lymphoma, and did not include patients with other subtypes of lymphoma, thus limiting the applicability of the results to the overall patient population with lymphoma. Thirdly, the study employed a prospective cross-sectional study design, and the assessment was conducted only at the beginning of the study. We need to consider the fluctuations in muscle mass and clinical parameters over time to guide the development of treatment strategies at different stages.

In conclusion, despite a high degree of heterogeneity between individual studies, it was found that ultrasonography is safe and feasible for the diagnosis of sarcopenia in patients with lymphoma. Particularly for fragile, elderly, and immobile patients, ultrasound is geographically and physically accessible, making it a valid tool for measuring sarcopenia. Assessment of sarcopenia can assist clinical physicians and dietitians in comprehensive evaluation of lymphoma patients, facilitating timely identification of conditions requiring early intervention and treatment, ultimately improving the clinical outcomes of patients. Future research can further explore the potential applications of ultrasound in patients with DLBCL. For instance, ultrasound can be employed to monitor treatmentrelated myotoxicity, predict treatment responses, and improve patient prognosis. Moreover, the integration of ultrasound with other biomarkers (such as creatine kinase and creatine) is expected to provide a more comprehensive perspective on muscle health assessment in DLBCL patients.
